# Liposuction-Like Sclerotherapy Technique for Microcystic Lymphatic Malformation

**DOI:** 10.7759/cureus.22795

**Published:** 2022-03-03

**Authors:** Huaijie Wang, Chong Xie, Weilong Lin, Jinbang Zhou, Weijia Yang, Zhengtuan Guo

**Affiliations:** 1 Department of Pediatric Surgery, Xi’an International Medical Center Hospital, Xi'an, CHN

**Keywords:** overgrowth, surgery, liposuction, sclerotherapy, bleomycin, lymphatic malformation

## Abstract

Background

The treatment for microcystic lymphatic malformation (LM) remains challenging. We describe the liposuction-like sclerotherapy technique, a new treatment for extensive microcystic LM.

Methods

LM data was retrospectively reviewed. This study included patients with a microcystic LM component treated by liposuction-like technique with bleomycin sclerotherapy.

Results

Between June 2016 and October 2019, 39 consecutive patients (male/female ratio: 21:18; mean age, 33.6 months; range: 5 months to 15 years) with microcystic LM were treated by liposuction-like sclerotherapy (LS-LS) technique. Fifty-six sessions of LS-LS were performed (mean of 1.44 sessions per patient; range: one to four sessions). Follow-up ranged 6-30 months (mean of 21 months). We observed no major complications. Transient minor complications included: postoperative noninfectious fever, vomiting, temporary skin edema, pigmentation, mild local depressions, and/or irregularities, and a small hyperpigmented scar at the incision. No postoperative infection, skin ulcer, or necrosis occurred. The patients’ symptoms were successfully resolved or stable. A sub-complete response and partial response were observed for 26 (76%) and 13 patients (33%), respectively.

Conclusion

The LS-LS technique for microcystic LMs is safe, feasible, and effective. This technique is an effective intervention with which it is possible to manage and potentially cure microcystic LM clinically.

## Introduction

Lymphatic malformations (LMs) are congenital lymphatic anomalies, which may result from abnormal lymphatics development, often with underlying somatic PIK3CA mutation [1]. LMs can be solitary or multifocal and can be classified into macrocystic (＞1-2 cm), microcystic (＜1 cm), or mixed cystic lesions [2]. Sclerotherapy is the mainstay treatment for macrocystic LMs but has a poor response in microcystic and mixed cystic lesions [3-5]. The microcystic lesion typically consists of multiple small cysts (＜1 cm), in which traditional sclerotherapy and direct injection are less effective due to the small size of the lumen. Recently, encouraging results were obtained in microcystic LMs (mLM) with intralesional bleomycin sclerotherapy [4-7]. In traditional percutaneous sclerotherapy, the cyst was accessed and then sclerosant was injected with or without imaging guidance. However, in cases with extensive microcystic component, it is difficult to obtain successful results despite multiple sclerotherapy sessions with traditional techniques. In the microcytic lesions, there is a relatively large amount of soft-tissue component compared to the small cysts. Therefore, even when the cysts are resolved or reduced there is a residual soft-tissue mass that cannot be treated with sclerosants. We have reported a new treatment, liposuction-like sclerotherapy technique (LS-LS) that successfully managed superficial LM in some cases [8]. Herein, we describe the treatment effect of this technique for mLM involving the trunk and extremities. Perioperative data and imaging were studied to evaluate the efficacy of this technique.

## Materials and methods

This review was carried out in accordance with the requirements of and after approval by the institutional ethics committee and Institution Ethics Review Board of Xi’an International Medical Center with approval number XIMED (2019-PedSurg-03). All parents were fully informed about this new and alternative nature of the techniques, including the potential side effects of bleomycin (fever, vomiting, allergy, changes of pulmonary function, etc.). Treatment was performed after obtaining written informed consent in all patients. Patients with extensive mixed cystic LM or microcystic LM of the trunk and extremities demonstrated on MRI treated by liposuction-like technique with bleomycin sclerotherapy were included. Patients with predominant macrocystic lesions were excluded from this study.

Diagnosis

The diagnosis of mLM component was based on clinical and imaging study of our multidisciplinary team, including pediatric interventional radiologists, sonography expert, and pediatric surgeon. The diagnostic criteria were based on clinical, sonographic, and MRI study, including clinical history, dermal vesicles, a soft-tissue cystic mass containing lymphatic fluid or hemorrhagic fluid, and fluid signal on MRI. Imaging was used to examine the extent and the architecture of the lesions. No preoperative biopsy was performed in this case series. Patients with only macrocystic LM were excluded.

Liposuction-like sclerotherapy technique

**Figure 1:**
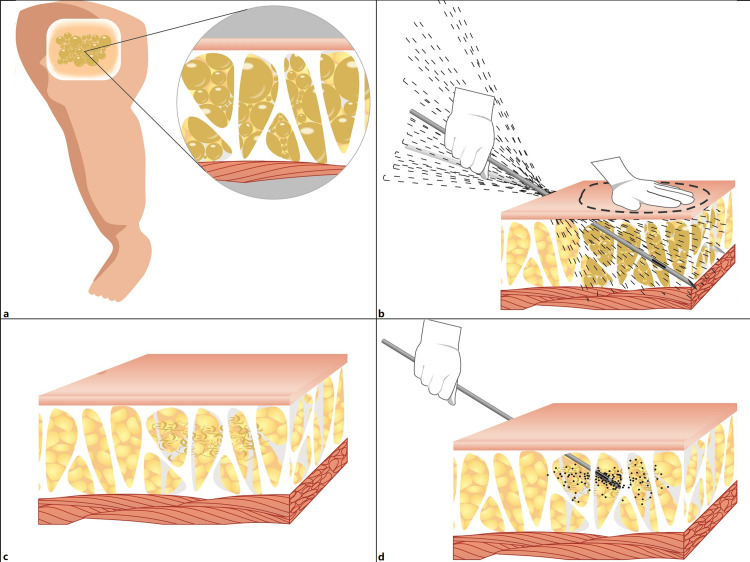
Operative technique of the liposuction-like sclerotherapy. Panel a: A microcystic lymphatic malformation of the lower extremity. Panel b: After topographic marking and tumescent infiltration, the vacuum-assisted liposuction-like operation was performed. Panel c: The wall of lymphatic malformation was destroyed after suction. Panel d: Showing the diffusion of the mixture of contrast medium and bleomycin in the treatment area.

**Figure 2:**
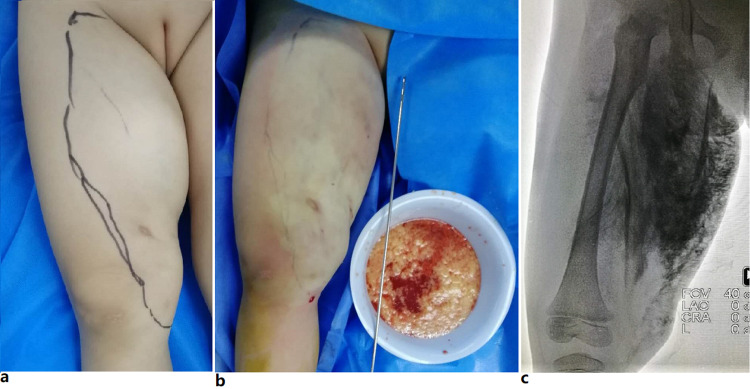
Microcystic lymphatic malformation. Photograph of the liposuction-like sclerotherapy process. Panel a: A microcystic lymphatic malformation of the right thigh. Topographic marking has been made before operation. Panel b: Immediate appearance after the liposuction-like operation was performed. Part of subcutaneous fat and lymph fluid was removed through the sharp, triple hole (Mercedes) cannula. Panel c: Showing the diffusion of the mixture of contrast medium and bleomycin in the treatment area.

**Figure 3:**
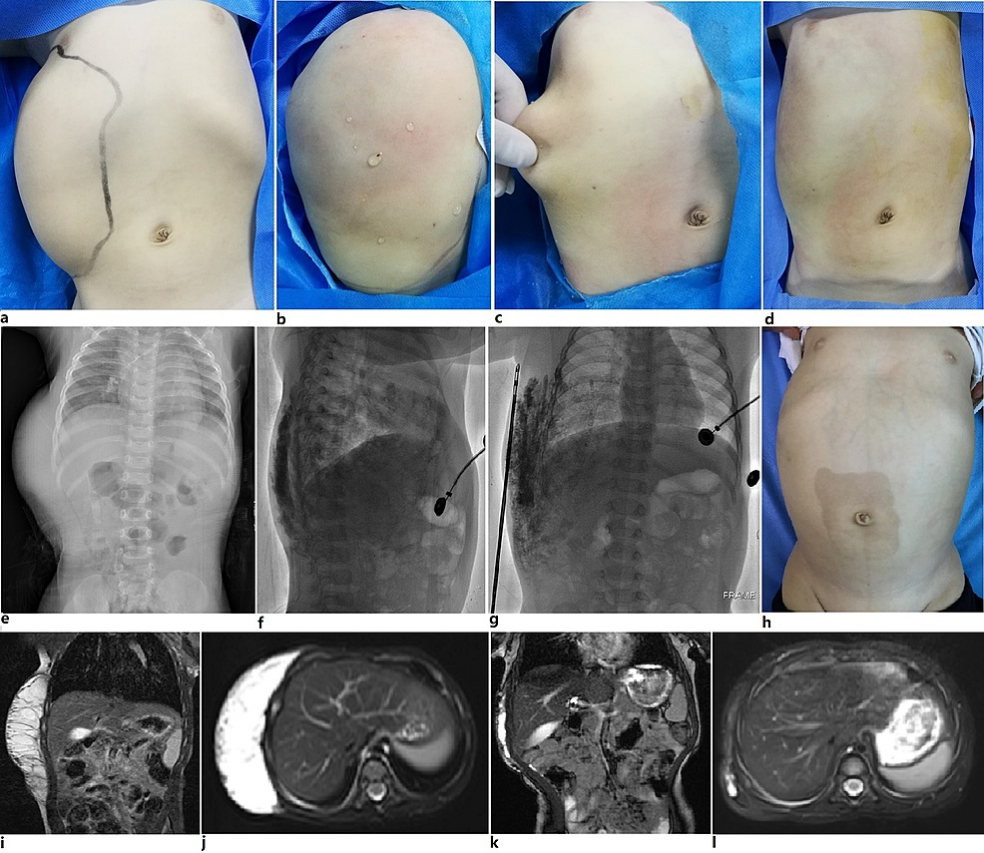
Mixed cystic lymphatic malformation. Details of the liposuction-like sclerotherapy on photographic and imaging view. Panel a, e, i, and j: An extensive mixed cystic lymphatic malformation of the right thoracic and abdominal wall. The position and extent of the lesion were topographically marked. Panel b: Showing the tumescent infiltrated lesion area. Panel c: During the vacuum-assisted liposuction-like operation, a pinch test was used to judge the endpoint. Panel d: At the endpoint of operation, the treatment area became smooth and flattened. Panel f and g: Showing the diffusion of the mixture of contrast medium and bleomycin in the treatment area, confirmed on lateral and frontal fluoroscopic imaging. A 2-mm in diameter, sharp, triple hole (Mercedes) cannulas in double row configurations were noticed on Panel g. Panel h: Showing the cosmetic appearance eight months after the operation. Panel k and l: Showing the MRI eight months after the operation. The result of treatment in this patient was graded as sub-complete response.

Preoperative topographic markings were made on the patient with permanent markers to delineate the position and extent of lesions by an ultrasonologist. The operation was performed under general endotracheal anesthesia in a hybrid or interventional operation room.. Patient positioning was planned to provide optimal exposure of treatment areas and the ability to assess lesions during operation. Like liposuction in body contouring and fat grafting, tumescent local anesthesia was used in this procedure [9]. Tumescent infiltration was performed in the lesion area according to the liposuction procedure. The tumescent solution used for operations consists of lidocaine, epinephrine, and saline (2% lidocaine 20 mL + 1:1000 epinephrine 1 mL + saline 1000 mL). Waiting at least 10 minutes after injection could ensure the optimal vasoconstrictive effect. The maximum dose for lidocaine in tumescent solution was limited to 35 mg/kg [9,10]. A small incision was made at the border of the treatment area rather than within the area. We used 2 mm in diameter, 15-30 cm in length, blunt/sharp, and triple hole (Mercedes) cannulas in single or double row configurations. Using the vacuum-assisted liposuction technique, part of the subcutaneous fat and lymph fluid of LM was removed. During aspiration, we feathered the periphery of a treated area also to cover the potential, nonvisible lymphatic lesions. In order to destroy all small cysts within the lesion, we used pinch test and sonographic exam to judge the endpoint when the treatment area became smooth and flattened. After completion of the aspiration, the bleomycin dilution was injected through 22-gauge needles directly inserted into the treatment area subcutaneously. Bleomycin was diluted with 5-10 mL of contrast medium (iodixanol injection) to obtain the mixture. The maximum dose for bleomycin was 1 mg/kg per session, injected in children (maximum 15 mg). At the end of the injection, a fluoroscopic image was obtained to check spontaneous diffusion of the mixture in the treatment area. Thereafter, the probe was used to dispense bleomycin. The liposuction process was redone without vacuum to obtain more even distribution and better infusion of the bleomycin dilution in the operated area. Finally, the diffusion of the mixture was rechecked by fluoroscopic imaging (Figures 1-3). Local compressive dressing over the small incision was applied for preventing bleomycin dilution overflow from the incision after completion of the operation.

Treatment details

The tumescent technique used in the LS-LS technique was the same as that in liposuction for body contouring and fat grafting. The volume of tumescent solution for this technique varied as the size of the lesion. We inject the tumescent solution regardless of whether the infiltration needles were intra- or pericystic placed. The endpoint of infiltration was the skin becoming pale. The dose of bleomycin used was 0.5-1 mg/kg per session (maximum 15 mg). The mean operation time was 50 minutes (range: 30-90 minutes).

Postoperative care and follow up

No antibiotics were applied perioperatively. During the first six hours, a cold compress was routinely applied to reduce excessive local edema and to delay the absorption of bleomycin by blood vessels. We instructed the patient or parents to observe operative complications such as skin pigmentation, ecchymosis, edema, or re-enlarging after discharge. One month later, patients came back to the outpatient room for complication evaluation. If no complication was observed, a monthly follow-up was performed between two treatment sessions. At the sixth-month follow-up, patients underwent an MRI study.

Two independent radiologists assessed the imaging to evaluate treatment response. The response of sclerotherapy was initially assessed by a six-month follow-up on MRI. On the archived MRIs of the picture archiving and communication system (PACS), we used self-contained tools to assess the treated lesion. Postoperative MRI responses were divided into sub-complete (＞80% of the lesion decreased), partial (25-80%), and no response (＜25%). Sclerotherapy was repeated if required. The final result of therapy was evaluated by our multidisciplinary consensus at the vascular anomalies center during follow-up.

## Results

Between June 2016 and October 2019, 39 consecutive patients with microcystic LM were treated by the LS-LS technique. The patient characteristics are summarized in Table 1.

**Table 1:** Summary of patients with microcystic lymphatic malformation treated with liposuction-like sclerotherapy technique.

Patient characteristics	
Age	5 months-15 years, mean 33.6 months
Sex (male: female)	21:18
The anatomic sites of microcystic lymphatic malformation	
Right thoracic and abdominal wall	n=8
Left thoracic and abdominal wall	n=7
Bilateral thoracic and abdominal wall	n=1
Left upper arm and thoracic wall	n=5
Left lower extremity	n=4
Right buttock	n=2
Left buttock	n=3
Right forearm	n=3
Right leg	n=3
Right thigh	n=2
Right upper arm	n=1
Transient minor complications	
Fever (>39.0℃) during the first 24 hours	n=8
Vomiting	n=3
Response	
Sub-complete response	n=26
Partial response	n=13

Three patients in this study presented with extensive mixed cystic LM with predominantly microcystic component involving the entire left lower extremity and buttock. Nine patients had a history of LM treatment before the LS-LS technique, five patients had a previous resection, and four patients had previous bleomycin and/or absolute ethanol sclerotherapy. The interval between previous treatments and the LS-LS technique was at least 6 months.

Fifty-six sessions of LS-LS were performed (mean of 1.44 sessions per patient; range: one to four sessions). The three patients, with extensive mixed cystic LM with predominantly microcystic component involving the entire left lower extremity and buttock, received three sessions of LS-LS. Some patients with an mLM of thoracic and abdominal wall underwent multiple sessions of LS-LS (two-four sessions) because the lesion was too thick or extensive to be fully tumescent infiltrated. There was a risk of excessive intra- and postoperative bleeding in unfully tumescent infiltrated areas. Therefore, the staged operation was performed in these patients. The mean follow-up was 21 months (range: 6-30 months).

Complications

No major complication was observed. Transient minor complications included: eight patients developed severe postoperative noninfectious fever (>39.0℃) during the first 24 hours, three patients experienced postoperative vomiting that resolved with nonspecific intravenous fluid administration within 24 hours (Table 1). Temporary skin edema, pigmentation, mild local depressions and/or irregularities, and a small hyperpigmented scar at the incision were noticed in all patients. Pigmentation over the treatment area gradually faded but persisted for at least months or more than a year without intervention. Excessive skin was observed in seven patients with too extensive lesions during follow-up. No postoperative infection, skin ulcer, or necrosis occurred.

MRI evaluation

**Figure 4:**
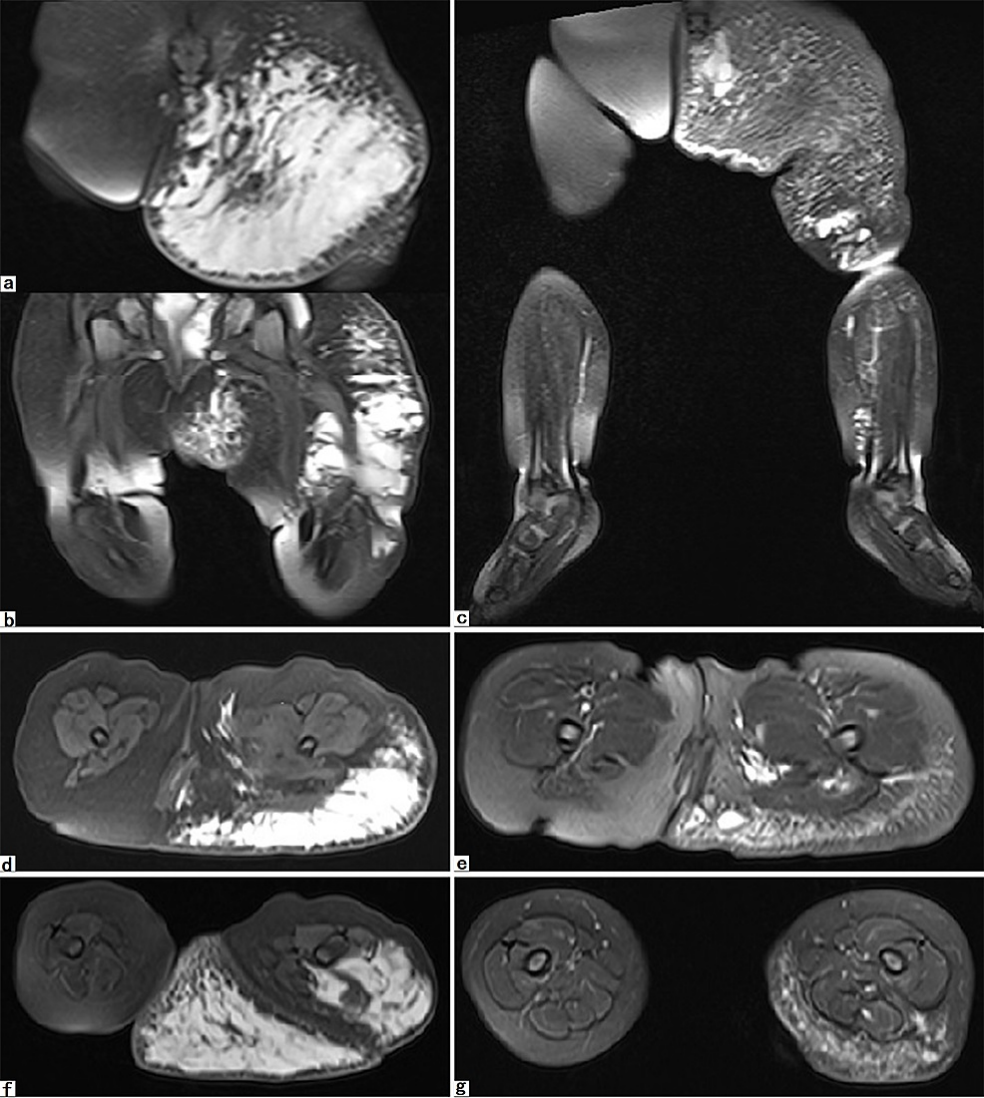
MRI of an extensive lymphatic malformation before and after treatment. Panel a, b, d, and f: An extensive mixed cystic lymphatic malformation with predominantly microcystic component involved entire left lower extremity and buttock. Panel c, e, and g: After 6 months following treatment, the lesion had largely decreased by imaging criteria. It was noticed that the obviously enlarged left buttock had a great improvement on MRI. Furthermore, a dramatic improvement in the intramuscular component was observed simultaneously, which implies interconnection with subcutaneous component. The result of treatment in this patient was graded as partial response.

Response (＞25% of the lesion decreased by imaging criteria) was observed on MRI for all patients (Figures 3-4). A sub-complete response was observed in 26 patients (67%), and partial response in 13 patients (33%) (Table 1). A minor or no change in the size of the lesion was not observed.

Clinical outcome of symptoms

The patients’ symptoms comprised dermal grouped hemorrhagic vesicles (n=2), obvious mass (n=7), pain (n=8), hypertrichiasis (n=1), and limb asymmetry in girth (subcutaneous mLM and fat thickened, (n=16)). During follow-up, local mass became flattened. Dermal grouped hemorrhagic vesicles also decreased. Pain (n=8) was successfully resolved. Hypertrichiasis was stable. Limb asymmetry in girth was successfully controlled responding to graded responses on MRI.

## Discussion

The mLM has been understood to be caused by somatic mutations in PIK3CA. These malformed lymphatics do not communicate with the normal lymphatic system around the tissues [1,11]. A treatment choice is partial surgery, but total resection is not always possible [11,12]. On the other hand, traditional percutaneous sclerotherapy techniques that the cyst is accessed and then sclerosant is injected are less effective for mLMs, partially due to the small size of the cysts or channels and poor diffusion of sclerosants within the lesion. And perhaps more crucial is that the extensive overgrowth of soft tissue is extremely difficult to manage. In order to disrupt the intactness of multiple or numerous small cysts, to improve the contact between sclerosants and cyst wall, and to remove the excessive fatty deposition simultaneously, we developed this new technique for the difficult mLMs, providing encouraging safety and efficacy outcome.

The following principles are speculated in the LS-LS technique:

The wall completeness of most cysts is interrupted by the cannula during the liposuction-like procedure, which can transform a cluster of small cysts into an irregular macrocystic-like lesion. Higher concentrations of the sclerosant can be achieved, a higher efficacy may be obtained. Most of the lymphatic fluid and tumescent solution is extracted by the LS-LS technique. The vasoconstrictive effect of tumescent solution may result in delayed absorption of the sclerosant by neurovascular bundles, ensuring increased contact time between the sclerosant and residual cyst wall.

The delivery of bleomycin into subcutaneous soft tissue can cause some damage to the normal tissue, but may not be so powerful to cause skin necrosis [5,13]. The latticework of neurovascular bundles left in-situ remains to nourish the overlying skin and associated adnexal structures. Also, small amounts of isosmotic nonionic contrast agent (iodixanol) used in our technique rarely cause skin necrosis and ulceration. In mLMs with traditional direct puncture technique, some cystic lesions were inaccessible, even under imaging guidance. Consequently, in traditional percutaneous sclerotherapy technique, the extravasation of bleomycin into the surrounding normal tissues is inevitable for mLM treatment. However, skin necrosis has been rarely reported following intralesional bleomycin injection for mLMs. Nevertheless, we did not observe skin necrosis in this case series.

The liposuction-like maneuver without negative pressure following sclerosant injection allows more even and deeper delivery of the sclerosant through a cannula-passed channel network. Extensive bleomycin distribution (relative low concentration of agent) can reduce the risk of subcutaneous fibrosis. After months of recovery, the skin of the treatment area became pinchable again in our series. Removing the excessive fat of a treated area also contributes to smoother contours. Based on the above principles and compared with traditional percutaneous sclerotherapy, the LS-LS technique achieves more extensive treatment area and ensures more efficient management for extensive lymphatic lesions. Several percutaneous techniques have been described for the treatment of microcystic lesions, including intralesional injection of sclerosant and lymphographic-like sclerotherapy with doxycycline, bleomycin, or OK-432 [4-7]. However, multiple sessions of therapy are often required, especially for extensive lesions with very small cysts. A larger cumulative dose of sclerosant was needed, with a higher risk of sclerosant-related complications [5].

The LS-LS technique described in this review has evolved from the conventional intralesional injection and resection procedure for macrocystic LMs. In the early years, we noticed the interconnection among cysts when open resection was performed for macrocystic and mixed LMs. This finding was confirmed by intralesional endoscopic examination and resection of LMs (unpublished data). Also, the simultaneous regression of mixed cysts was observed following a simple injection of bleomycin into the macrocystic component. Moreover, we performed fluoro-guided imaging for mLMs, and confirmed the interconnection among microcysts (unpublished data). These findings have been reported recently by other authors [7]. Therefore, this interconnection-based infusion sclerotherapy may be an effective treatment technique for mLMs. However, this technique is probably less effective for extensive mLM treatment with tissue overgrowth. In order to enhance the interconnectivity among cysts and remove overgrew tissue to manage extensive mixed and microcystic LMs more efficiently, it was decided by our team to introduce the tumescent liposuction technique in 2016. Therefore, we have developed the LS-LS technique in recent years.

There are some limitations to our study. This study was a retrospective review of the case series and was not adequately convincing since only patients with microcystic components were enrolled. It is difficult to compare outcomes of the LS-LS technique with those in previous publications because various criteria of response evaluation are frequently used. It now seems that the LS-LS is inapplicable to mLMs of the anterior cervical region and face, because of neighboring vital structures and the complexity of facial anatomy. Nevertheless, our study confirmed the value of this minimally invasive technique for debulking mLM lesions of the trunk and extremities, without severe complication. Prospective comparison of the LS-LS with other managements is expected to obtain more convincing results.

## Conclusions

The treatment of mLMs remains challenging, and a new therapeutic technique is required. We report a new treatment technique for these lesions. The LS-LS technique for mLMs is safe and effective, especially for extensive lesions. Prospective studies comparing traditional percutaneous sclerotherapy with this new technique are warranted.
